# How In-Group Bias Influences Source Memory for Words Learned From In-Group and Out-Group Speakers

**DOI:** 10.3389/fnhum.2019.00308

**Published:** 2019-09-12

**Authors:** Sara Iacozza, Antje S. Meyer, Shiri Lev-Ari

**Affiliations:** ^1^Max Planck Institute for Psycholinguistics, Nijmegen, Netherlands; ^2^International Max Planck Research School for Language Sciences, Nijmegen, Netherlands; ^3^Radboud University Nijmegen, Nijmegen, Netherlands; ^4^Department of Psychology, Royal Holloway, University of London, Egham, United Kingdom

**Keywords:** in-group bias, novel word learning, source memory, decision bias, lexical representations

## Abstract

Individuals rapidly extract information about others’ social identity, including whether or not they belong to their in-group. Group membership status has been shown to affect how attentively people encode information conveyed by those others. These findings are highly relevant for the field of psycholinguistics where there exists an open debate on how words are represented in the mental lexicon and how abstract or context-specific these representations are. Here, we used a novel word learning paradigm to test our proposal that the group membership status of speakers also affects how speaker-specific representations of novel words are. Participants learned new words from speakers who either attended their own university (in-group speakers) or did not (out-group speakers) and performed a task to measure their individual in-group bias. Then, their source memory of the new words was tested in a recognition test to probe the speaker-specific content of the novel lexical representations and assess how it related to individual in-group biases. We found that speaker group membership and participants’ in-group bias affected participants’ decision biases. The stronger the in-group bias, the more cautious participants were in their decisions. This was particularly applied to in-group related decisions. These findings indicate that social biases can influence recognition threshold. Taking a broader scope, defining how information is represented is a topic of great overlap between the fields of memory and psycholinguistics. Nevertheless, researchers from these fields tend to stay within the theoretical and methodological borders of their own field, missing the chance to deepen their understanding of phenomena that are of common interest. Here, we show how methodologies developed in the memory field can be implemented in language research to shed light on an important theoretical issue that relates to the composition of lexical representations.

## Introduction

Previous findings have shown that people utilize any cue they have available (e.g., gender, social class) to establish whether or not others are members of their own in-group (e.g., Bargh et al., 2012). Group membership can affect how people process and remember information related to those others, with in-group information receiving more attention and being better remembered than out-group information (Hugenberg et al., 2010; Greenstein et al., 2016). While advantages for in-group members have been reported to affect a wide range of cognitive phenomena (see Xiao et al., 2016; Molenberghs and Louis, 2018 for reviews), they have not been directly tested in the context of language processing and language learning, yet.

Such effects are relevant for models of language processing because they have consequences for an ongoing debate on how words are represented in the mental lexicon. One aspect of this broad issue is how well listeners maintain information that is not strictly linguistic but that relates to the context, such as the social identity of the speaker producing a word. In the memory literature, this type of information is referred to as source memory and it is a topic that has been extensively studied. By using memory tests developed to probe source memory, researchers in the field of psycholinguistics can gain a better understanding of how speaker-related information is encoded in the representations of words and whether the encoding of such information is modulated by social factors, such as the group membership status of the speakers.

The aim of the current study is to investigate the proposal that in-group biases permeate language processing as well, and that they affect the level of detail of speaker-related information that is encoded when learning new words. We propose that representations of words learned from in-group members are more likely to contain highly specific speaker-related information, as compared to representations of words learned from out-group members, and that such differences are in turn influenced by how strongly each learner prefers their in-group members over out-group members.

Before turning to the current study, we review the relevant literature. We start by reporting evidence that shows that the social identity of the speaker affects how listeners process language. We then describe existing exemplar-based theories of language processing that provide a theoretical framework for understanding effects of speaker identity on language processing. We then point to a potential limitation of these models, namely, their tendency to assume that the speech of all speakers is treated equally. We propose that existing models should integrate parameters that allow different degrees of encoding specificity and assigning different weight to linguistic input depending on speaker group membership status. Specifically, we propose that linguistic information provided by in-group speakers is encoded in more detail than information from out-group speakers. We motivate our proposal with evidence from non-linguistic studies in social psychology that report group membership effects on memory and information processing.

Previous research indicates that when interacting with others, information about their social identity is rapidly extracted (see Bargh et al., 2012, for a review) and can influence people’s attitudes and preferences toward those others (e.g., Greenwald and Banaji, 1995; Jones and Fazio, 2010; Kinzler et al., 2011). There exists diverse evidence showing that others’ social identity can influence how listeners process language. For instance, it has been shown that, when a speaker’s social identity is made available via the speaker’s voice, listeners take the identity into consideration and have particular expectations about what will likely be said. If these expectations are not met, such as when the desire of looking like Britney Spears is reported in a man’s voice, language processing becomes harder (Van Berkum et al., 2008, see also Walker and Hay, 2011; Martin et al., 2016). Similarly, speaker social identity can affect how listeners perceive speech sounds (e.g., Johnson et al., 1999; Niedzielski, 1999; Hay et al., 2006a, b). For example, changing listeners’ expectations of a speaker’s place of residence affected their responses in a diphthong identification task. Participants reported hearing what they believed to be more representative of the supposed speaker’s linguistic community, independent of the actual linguistic input, which was identical across the two conditions (Niedzielski, 1999). This suggests that information about the speaker affects speech perception.

In short, this body of evidence shows that information related to the speaker’s identity is extracted along with the linguistic input and can influence the processing of the latter. Existing exemplar-based models of speech processing argue that the reason that social information is used in language processing is because it is encoded along with linguistic input. These models state that linguistic experiences are encoded as rich episodic memories (i.e., exemplars) (e.g., Hay et al., 2006a; Goldinger, 2007; Nielsen, 2011; see Drager and Kirtley, 2016, for a review) that contain information which is both language-specific (e.g., includes phonetic, lexical, and syntactic details) and context-specific (e.g., includes pragmatics, speakers’ characteristics) (e.g., Drager and Kirtley, 2016).

Recently, in a new model by Münster and Knoeferle (2018) the contributions of encoding speakers and listeners’ characteristics during on-line language processing were formally defined. Grounded on a large body of empirical evidence, the model posits that comprehending language in context, by, for instance, extracting both speaker-specific and language-specific input in tandem, may speed up and/or ease comprehension. For example, consider a scenario in which the utterance “Every evening I drink some wine before I go to sleep” is produced by an adult speaker. Based on the age of the speaker, listeners can build up probabilistic inferences about what is more likely to follow the verb *drink* (e.g., in the case of an adult, the word *wine* is more probable than the word *milk*). By pre-activating lexical items that are more probable, listeners can easily make sense of the new piece of information, i.e., the word wine, speeding up comprehension (see Münster and Knoeferle, 2018 for details).

Crucially, Sumner et al. (2014) proposed that the social context might not only be encoded with the linguistic input but might modulate the strength of its encoding. In support of this account, Sumner et al. (2014) showed that idealized phonetic variants are encoded with greater weight than common, therefore more frequent, phonetic variants. According to their model, phonetic variants with higher prestige (i.e., idealized ones) receive an advantage in representation and processing as compared to variants characterized by lower prestige. Extending their theory to more general linguistic processes, one could hypothesize that people would encode linguistic variations more strongly if they are associated with contexts and speakers that have a special status.

Here, we propose that learning new words from speakers that are ascribed a special status might lead to lexical representations that are richer in contextual information (e.g., speaker-related information), as compared to representations of words learned from speakers without a special status. An example of speakers that are ascribed a special status is the case of in-group members. Indeed, there is evidence suggesting that group membership influences input processing and learning. For instance, memory is usually better for in-group faces than out-group faces (e.g., Van Bavel et al., 2008; Hugenberg et al., 2010) and for information delivered by in-group than by out-group members (e.g., Frable and Bem, 1985; Wilder, 1990). Furthermore, people learn better and process more quickly new associations between previously neutral stimuli (e.g., geometrical shapes) and in-group membership (e.g., the logo of their favorite football club) than associations involving out-group membership (Moradi et al., 2015; Enock et al., 2018).

One way in which in-group biases may work is via the recruitment of additional cognitive resources (Meissner et al., 2005; Van Bavel and Cunningham, 2012). Such additional resources have been suggested to lead to in-group representations that are characterized by a higher level of detail than out-group representations. For example, when processing in-group related information, people were shown to encode the source of information in more detail than when the information was related to out-group members. This resulted in them being better in a source memory task when identifying in-group sources than out-group sources (e.g., Greenstein et al., 2016), suggesting that being exposed to in-group membership boosts the encoding of individual-specific information (see Hugenberg et al., 2010, for a similar account).

No study has tested whether lexical representations for the same words can depend on the identity of the speaker that tends to use them. If this is the case, this will have implications for language learning, language processing, and linguistic representations. It would extend current theories that examine the role of input and its distribution in language acquisition and representation by showing that the same distribution can have different effects depending on who are the speakers that provide different tokens in the input. As a first step, the current study was designed to investigate which social information learners encode when they learn new words from speakers who either belonged to the learners’ social group (i.e., in-group members) or did not do so (i.e., out-group members).

### The Current Study

We hypothesize that listeners encode the social identity of the speakers from whom they learn novel words and that the social identity influences how detailed speaker-specific information is encoded. To test these predictions, we carried out the current study in which we examined participants’ source memory for words learned from speakers from different social groups. In the Main Experiment, participants were exposed to a learning context in which they learned new words from speakers who supposedly shared their university affiliation (i.e., in-group speakers) and from speakers with a different affiliation (i.e., out-group speakers). In the Control Experiment, participants learned from two groups of speakers who supposedly attended two foreign universities. Since in the Control Experiment the group membership was not manipulated, because both universities were unrelated to the participants, we could check that the patterns hypothesized to be found in the Main experiment were indeed a reflection of the social saliency ascribed to speakers’ group membership and not simply a consequence of the contrastive nature of our manipulation (i.e., teaching competing labels spoken by different groups of speakers).

During the word learning task, all participants in both experiments learned novel labels for uncommon gadgets. Crucially, target gadgets received two competing but equally fitting labels, one from a speaker of each affiliation (e.g., *citrus-peller* vs. *citrus-schiller*, in English lemon peeler vs. lemon stripper). Afterward, source memory for these words was tested in a recognition memory test. Participants were shown one speaker and one label at a time and asked if the speaker had produced the label in the previous phase (i.e., forced choice: yes/no). Lastly, we collected participants’ implicit in-group bias (see “Materials and Methods” section for details).

In the Main experiment, we predicted that participants would spontaneously monitor the speakers’ group membership status. Consequently, when asked to recognize the source of the new words, we expected participants to remember speaker social group but to struggle remembering the exact speaker that produced each word. Therefore, they should be more likely to misattribute words to incorrect speakers within the same affiliation than between different affiliations, i.e., there should be source memory confusion. Following our hypothesis about different levels of detail depending on social salience and group membership of the speakers, we predicted that words learned from in-group speakers would contain a higher level of detail about who produced them, compared to words learned from out-group speakers. This would result in in-group linguistic representations that are more speaker-specific and less prone to source memory confusion than out-group representations. Crucially, this in-group advantage should be stronger for participants exhibiting stronger in-group bias. This pattern is expected to result in a significant interaction involving speaker group membership and individual in-group bias. In the Control Experiment, we expected no differences between the two speaker affiliations. This would show that differential processing of information learned from different groups is specific to cases where group membership is socially salient.

## Materials and Methods

### Participants

One-hundred-twenty-four native Dutch speakers (age range: 18–26 years) participated in the study after providing their informed consent, as approved by the Ethics committee of the Social Sciences department of the Radboud University Nijmegen (project code: ECSW2014-1003-196). All participants were students or recent graduates of Radboud University Nijmegen. All participants were female, as were the speakers from whom they learned the labels. This was done to avoid that an additional social dimension (i.e., gender) of in-group status could interact with the one we manipulated (i.e., academic affiliation). Participants were randomly assigned to either the Main Experiment (*n* = 62) or the Control Experiment (*n* = 62).

### Materials

#### Materials for the Word Learning Task

##### Speakers

Eight fictitious speakers were created by pairing female faces selected from the Chicago Face Database (Ma et al., 2015) with the voices of native Dutch female speakers recorded in our laboratory. Prior to the experiment, voices were matched for perceived typicality and attractiveness (paired *d*-tests, ps > 0.05) via a norming on-line survey in which twenty different participants participated. Each speaker was a unique combination of one face and one voice, consistent across participants. Speakers’ academic affiliation was randomized across participants and indicated by the logo of the supposed affiliation displayed underneath the photo.

##### Affiliation logos

For the Main Experiment, original-color pictures of the logos of the Radboud University Nijmegen (i.e., in-group affiliation) and the ROC Nijmegen (i.e., out-group affiliation) were used. For the Control Experiment, original-color pictures of the logos of Pisa and Florence universities were used.

##### Objects and labels

Twenty-four images of unfamiliar gadgets (e.g., lemon peeler) and their corresponding labels were selected via a norming study (see Supplementary Appendix 1 for details). Half of the gadgets, hereinafter referred to as target gadgets, were presented with two competing labels, which were equated for goodness-of-fit and frequency. The other 12 gadgets were presented with a single label and served as fillers. All labels were produced by each speaker and audio-recorded.

#### Materials for the Individual In-Group Bias Task

##### Affiliation logos

The same logos used in the word learning task were used here.

##### Geometrical shapes

Black shapes for triangle, square, and circle were used.

### Procedure

#### Word Learning Task

**FIGURE 1:**
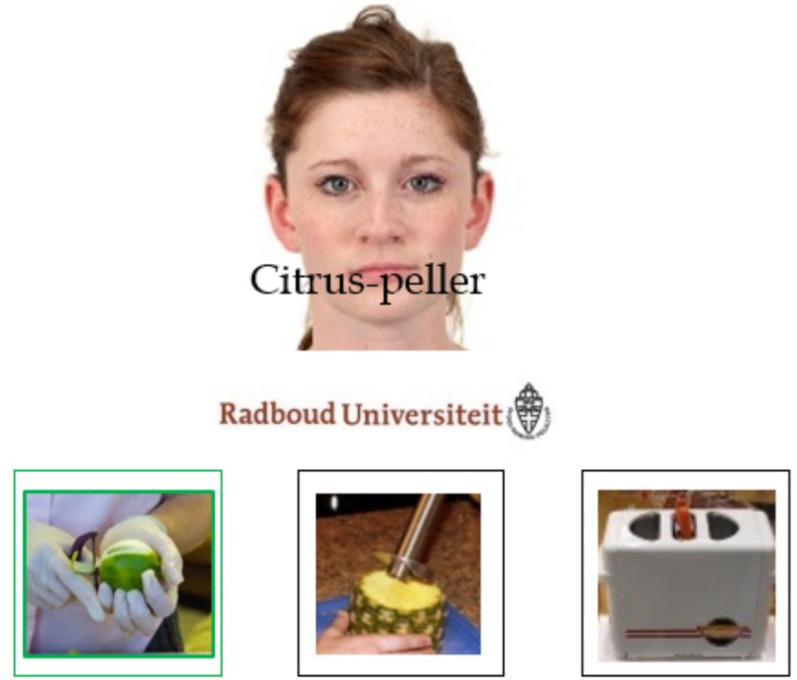
Example of the learning display. Participants had to select the gadget that was mentioned. In this case, they had to select the first image. Stimuli are not drawn to scale.

The word learning task consisted of an exposure phase and a test phase. The exposure phase was presented as a communication task in which participants were instructed to pay attention to all the stimuli presented (i.e., faces, gadgets and labels) and select gadgets based on what the speakers said, with no explicit reference to the academic logos. Participants saw 24 gadgets, each named by speakers of both groups. Half were target gadgets, for which the two groups of speakers provided competing, but equally fitting, labels, whereas the other half were fillers, for which unique labels were provided. Fillers were included to minimize participants’ awareness of the nature of the experimental manipulation (i.e., the contrastive nature of the labels). Note that not all speakers referred to all the gadgets. In fact, each gadget was only labeled by two of the eight speakers (one per group of speakers). Speaker group affiliation, speaker-label pairing, and label-group affiliation pairings were fully randomized per participant. On each trial, a photo of a speaker, together with the corresponding affiliation logo, was displayed (800 ms). Then, while the photos of speaker and logo were still on screen, the audio-recording related to the gadget label was played. Simultaneously, the written form of the label was superimposed upon the speaker’s mouth (1500 ms). Next, three gadgets appeared on the screen and participants selected the one that fit the audio and the written label (see Figure 1 for an example of the learning display^1^). If the response was wrong, the audio was repeated. Two exposure blocks were administered with half of the gadgets (i.e., six fillers and six targets) introduced in the first block, and the other half introduced in the second block. The gadgets were randomly allocated in the first or second exposure block per participant. Three exposure rounds were administered per block so that each display was repeated three times, once per round, in a randomized trial order.

**FIGURE 2:**
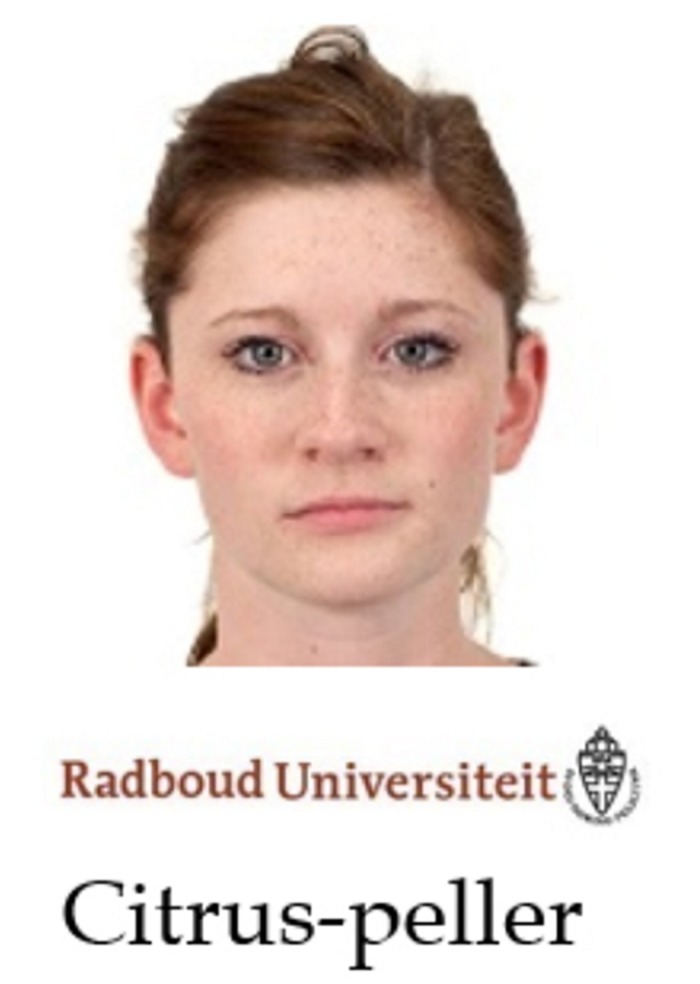
Example of a memory test trial. Participants indicated if the speaker had produced the label in the exposure task. Stimuli are not drawn to scale.

After each exposure block, participants performed a surprise source-memory recognition test on the gadgets introduced in the preceding exposure block only. In each trial, they saw a photo of a speaker with their affiliation logo and a written label (see Figure 2). Participants indicated whether the speaker had produced the label in the previous exposure phase via key press (i.e., forced choice: yes/no). Decisions were self-paced. Across the two memory test blocks, there were 288 trials in which all possible speaker-label pairings were shown. Of those 288 trials, 96 were filler-related trials (subsequently excluded from the analyses) and 192 were trials in which target gadgets were shown. Of the 192 target-gadget trials, 24 were matching trials (i.e., the speaker had indeed produced the label) and 168 mismatching trials (i.e., the label had not been used by the speaker). Of those mismatching trials, 72 were within-affiliation mismatching trials (showing a label along with a wrong speaker from the same affiliation as the correct one), 72 were between-affiliation mismatching trials (showing a label a speaker from the wrong affiliation). The remaining 24 trials showed a speaker with a label that competed with the one she used (e.g., the speaker that had used “citrus-schiller” was displayed with “citrus-peller”). They were only included to make all possible speaker-label combinations available, but they were not analyzed. Note that in all mismatching trials, the correct answer was that the pairing was incorrect because the speaker depicted in the photo had not used the displayed label in the exposure task.

#### Implicit In-Group Bias Task

Participants’ individual in-group bias was measured in a perceptual matching task (Moradi et al., 2015), which has been shown to provide results that are reliable within individuals and across different test sessions (Stolte et al., 2017). Three geometric shapes (circle, square, triangle) were randomly paired with logos of three academic affiliations. For the Main experiment, the logos depicted the in-group university – the Radboud University Nijmegen, and two out-group affiliations – the ROC Nijmegen and Tilburg University. To keep the two experiments comparable, participants in the Control experiment performed the task with logos of the Italian universities that appeared in the word learning task (Pisa and Florence) and a third Italian university, Bologna. Each association was initially presented ten times. Then, participants performed a practice block of 24 trials, followed by two blocks of 120 experimental trials each. In both practice and test trials, a fixation cross (500 ms) preceded a blank screen (between 1000 and 2000 ms) and the simultaneous presentation of logo and shape (600 ms), following the timings utilized in Moradi et al. (2015). Participants had 1500 ms to judge the accuracy of the pairing. Feedback was given only during practice. In-group bias in this task is usually indexed by faster and more accurate responses for stimuli that are newly associated with in-group membership compared to stimuli associated with out-group membership (e.g., Moradi et al., 2015).

## Results

All analyses were performed with mixed-effects modeling as implemented in the lme4 package (version 1.1-15; Bates et al., 2014) in R (R Core Team, 2016) and the models’ random structures were determined following the procedure suggested by Bates et al. (2015).

Before turning to the main analyses from the source memory test, we performed a sanity check to confirm that, at the group level, participants in the Main experiment showed the expected in-group bias in the perceptual matching task used to extract individual in-group bias measures.

### Group-Level In-Group Bias

#### Analyses Over RTs

Prior to analyses, trials with incorrect responses or with RTs faster than 200 ms or slower than 2100 ms were excluded. For these sanity-check analyses, we selected only matching trials (i.e., in which the logo of the university was displayed with the associated geometrical shape) which referred to the in-group university and the out-group university used in the study (i.e., the ROC Nijmegen). We then performed an outlier removal procedure by removing trials with RTs 2.5 SDs or higher from the mean per condition, per participant. The resulting dataset was analyzed using linear mixed-effect model in which log(10)-transformed RTs were predicted by the fixed effect for Group Membership (In-group vs. Out-group, reference level: In-group). We added per-participant random intercept and by-participant random slope for Group Membership. Results confirmed the usual patterns for this task: participants were faster at recognizing in-group-related associations than out-group-related associations (in-group: mean = 709 ms, SD = 212 vs. out-group: mean = 754 ms, SD = 199; β = −0.01, SE = 0.003, *t* = −5.03, *p* < 0.0001).

#### Analyses Over Accuracy

As with the RT analysis, the analysis included only matching trials (i.e., trials in which the logo of the university was displayed with the associated geometrical shape) which referred to the in-group university and the out-group university used in the study (i.e., the ROC Nijmegen). Accuracy was analyzed using a logistic mixed-effect model with a fixed effect for Group Membership (In-group vs. Out-group, reference level: In-group). We added per-participant random intercept and by-participant random slope for Group Membership. Results confirmed the usual patterns for this task: participants were better at recognizing in-group-related associations than out-group-related associations (in-group: mean = 94.70%, SD = 22.4 vs. out-group: mean = 92.88%, SD = 25.72; β = 0.4, SE = 0.1, *t* = 3.17, *p* < 0.01).

The analyses confirmed that in the Main experiment, at the group-level, participants showed a strong in-group bias for their own university. Successively, we extracted individual measures of in-group bias by calculating a per-participant measure of effect size, namely Cohen’s *d*, from both accuracy and RTs over in-group versus out-group matching trials. The measure calculated over RTs was not a significant predictor in any of the models we ran; thus, we will focus on the measure derived from accuracy.

Next, the results from the Main and Control experiments are presented separately because the in-group vs. out-group contrast only applies to the former experiment. The data from each experiment was analyzed following the outlined steps: (1) planned analyses on matching and mismatching trials, separately; and (2) *post hoc* analyses over d-prime and response bias values.

### Main Experiment

After each exposure round in the word learning task, participants were tested with a recognition memory test. In this test, they were presented with matching or mismatching speaker-label pairings and had to decide via key press if the label had or had not been produced by the speaker. We carried out analyses over matching and mismatching trials separately. We predicted that participants would show more accurate source memory of in-group labels, as compared to out-group labels, and that such advantage would be modulated by participants’ own in-group biases.

#### Matching Trials

To test whether source memory was better for in-group than for out-group words, we ran a logistic mixed effects model with accuracy as the dependent measure and fixed effects for Group Membership (In-group vs. Out-group, reference level: In-group), In-group Bias (centered continuous predictor), and their interaction. Block (Block1 vs. Block2, reference level: Block1) was included as covariate to control for potential confounds.^2^ We added per-participant and per-items random intercepts and a by-participant slope for Group Membership.

**FIGURE 3:**
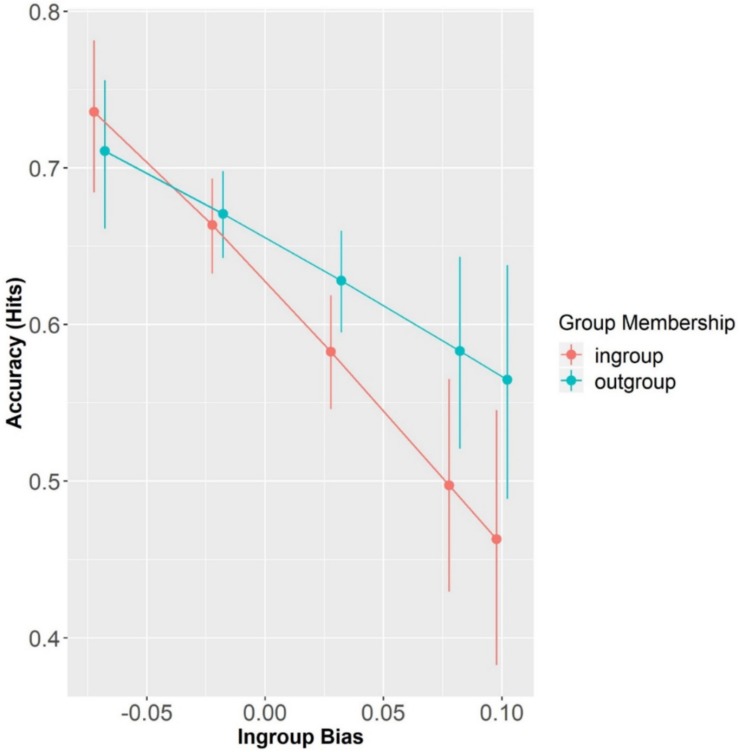
Accuracy (Hits) as a function of Group Membership and In-group Bias (centered). Error bars represent standard errors.

Overall, participants’ accuracy in the matching trials was 63.08% (SD = 48.28) and above chance level, as confirmed by a one-sample *t*-test (i.e., 50%) (*t* = 10.41, *p* < 0.001). Results showed that neither Group Membership (β = 0.10, SE = 0.13, *z* = 0.75, *p* = 0.45) nor its interaction with In-group Bias significantly predicted accuracy (β = 3.13, SE = 3.23, *z* = 0.97, *p* = 0.33). Participants’ accuracy did not differ between Block1 and Block2 (β = 0.02, SE = 0.11, *z* = 0.19, *p* = 0.34). However, participants’ In-group Bias significantly predicted accuracy, but only at the reference level, i.e., in-group membership (β = −6.90, SE = 3.17, *z* = −2.18, *p* < 0.05). By re-leveling Group Membership with Out-group as the reference level, we saw that accuracy for out-group speaker-label pairs was not modulated by the individual measure of In-group Bias (β = −3.76, SE = 2.93, *z* = −1.29, *p* = 0.20) (see Figure 3). This means that the more in-group biased participants were, the less accurate they were at recognizing speaker-label pairs, in particular when the speaker-label pairs were of their in-group.

#### Mismatching Trials

To test whether speaker group membership influenced the level of detail for speaker-specific information encoded with the new words, we analyzed accuracy on mismatching trials. By looking at participants’ performance on within-affiliation mismatching trials, where labels were paired with incorrect speakers but belonging to the same affiliation as the correct source, we were able to test whether the source-related information for novel words was speaker-specific (participants should have rejected the wrong source) or group-specific (participants would have incorrectly accepted the wrong source). We hypothesized that people would encode more speaker-specific information with in-group labels than with out-group labels. We therefore predicted greater confusion among out-group speakers than among in-group speakers in the within-affiliation mismatching trials. We also predicted that this difference in accuracy would depend on individual In-group Bias, such that the greater In-group Bias participants exhibited, the greater difference they should show between in-group vs. out-group trials. Conversely, in between-affiliation mismatches (i.e., where an in-group label was shown with out-group members, and vice versa) no differences were expected.

To test these hypotheses, we ran a logistic mixed model analysis with fixed effects for Mismatch Type (Within- vs. Between-affiliation, reference level: Within), Group Membership (In-group vs. Out-group, reference level: In-group), In-group Bias (centered continuous measure), and their interaction terms. We added Block as covariate, per-participant and per-item random intercepts and by-participant slopes for Group Membership and Mismatch Type.

**FIGURE 4:**
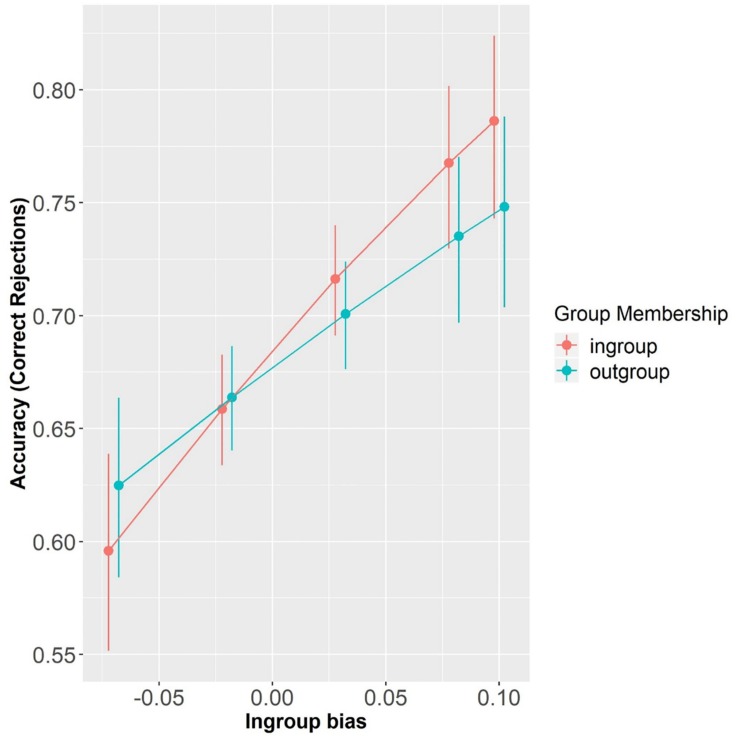
Accuracy (Correct rejections) as a function of Group Membership and In-group Bias (centered). Error bars represent standard errors.

Overall, participants’ accuracy on mismatching trials was 65.79% (SD = 47.45) and above chance level (i.e., 50%), as confirmed by a one-sample *t*-test (*t* = 31.31, *p* < 0.001). As expected, participants were more accurate for between–affiliation mismatches than for within–affiliation mismatches (β = 0.53, SE = 0.14, *z* = 3.10, *p* < 0.0001; mean = 70.35%, SD = 45.68 and mean = 61.22%, SD = 48.73, respectively). This shows that participants encoded speakers’ affiliations. Due to a practice effect, they were also more accurate in Block2 than in Block1 (β = 0.79, SE = 0.05, *z* = 15.95, *p* < 0.0001; mean = 73.61%, SD = 44.08 and mean = 58.09%, SD = 49.35, respectively). Participants’ performance was also significantly predicted by In-group Bias at the reference levels (β = 7.98, SE = 3.03, *z* = 2.64, *p* < 0.01) and by a marginally significant interaction of In-group Bias with Group Membership (β = −3.52, SE = 1.96, *z* = −1.80, *p* = 0.07), which suggests that participants with different strengths of In-group Bias were differently affected by speaker Group Membership. Specifically, simple effect analyses revealed that the larger the In-group Bias, the better participants were at correctly rejecting pairings involving the in-group membership (β = 7.98, SE = 3.03, *z* = 2.64, *p* < 0.01). On the other hand, participants’ In-group Bias did not predict their performance with pairings involving the out-group membership (β = 4.46, SE = 2.78, *z* = 1.6, *p* = 0.11) (see Figure 4).

Furthermore, neither the two-way interaction between In-group bias and Mismatch Type (β = −5.21, SE = 3.48, *z* = −1.5, *p* = 0.13), nor the three-way interaction between Mismatch Type, Group Membership, and In-group Bias reached significance (β = 3.10, SE = 2.54, *z* = 1.22, *p* = 0.22). Therefore, participants’ performance in both between- and within-affiliation mismatches was comparably affected by the Group Membership × In-group bias interaction.

In short, results from the matching trials revealed a *negative* relationship between In-group Bias and response accuracy, especially for in-group pairings. This pattern suggests that participants with stronger in-group bias were more likely to produce misses with in-group speaker-label pairs. On the other hand, results from the mismatching trials revealed a *positive* relationship between In-group Bias and accuracy, meaning that those strongly biased participants also produced fewer false alarms when in-group pairings were involved. These seemingly contradictory results can be reconciled by stepping away from simple accuracy analyses and by relying on signal detection theory measurements which capture detection sensitivity (namely, d-prime) and response bias (namely, C).

#### D-Prime and C Values

Analyses over d-prime and C measures allow us to test whether participants’ sensitivity and response bias during decision making processes differed for in-group vs. out-group related decisions. We calculated two d-prime values and two C values per participant for in-group and out-group trials separately. In order to generate values that reflected participants’ decisions to purely in-group or out-group trials, d-prime and C values were calculated from participants’ performance in matching trials (i.e., hit rates) and within-affiliation mismatching trials (i.e., false-alarm rates).^3^ Between-affiliation mismatches were not considered for these analyses because they were created by having an element (either label or speaker) from each group and were therefore not purely in-group or out-group related. We ran two linear mixed-effect models with either d-prime or C values as the dependent variable and Group Membership (In-group vs. Out-group, reference level: In-group), In-group Bias and their interaction as fixed effects. The models included per-participant random intercepts.

The model that explored the relationship between individual d-prime and the independent variables showed no significant main effects or interactions (ps > 0.57), suggesting that participants’ sensitivity was not modulated by speaker Group Membership or their own In-group Bias, nor the interaction between them (see Figure 6A).

**FIGURE 5:**
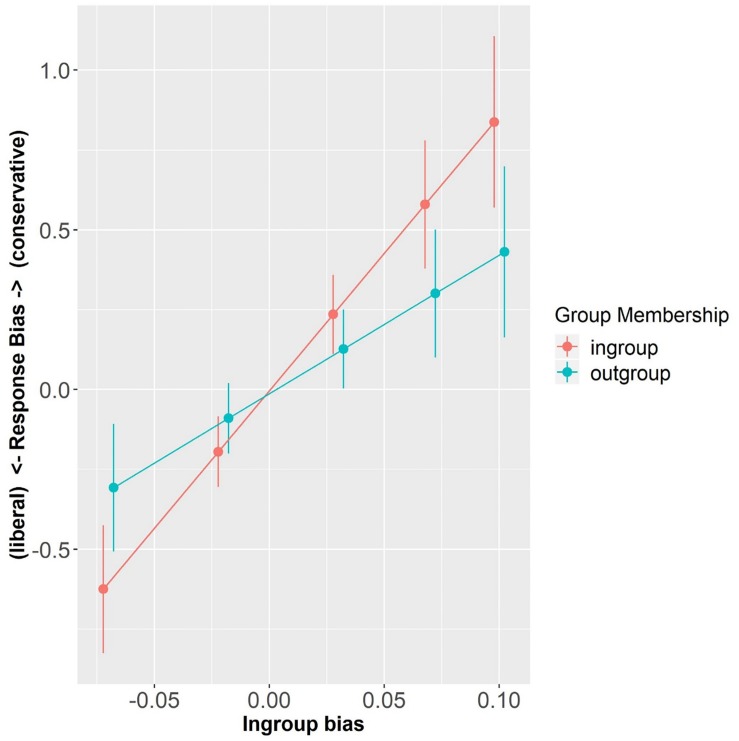
Response bias as a function of Group Membership and In-group Bias (centered). Error bars represent standard errors.

On the other hand, the model exploring C values showed a significant main effect of In-group Bias (β = 8.60, SE = 2.55, *t* = 3.38, *p* < 0.001) so that the more in-group biased, the more conservative participants were in their decision (i.e., having a bias for “no” responses). Importantly, there was a significant interaction between In-group bias and Group Membership (β = −4.26, SE = 1.97, *t* = −2.16, *p* < 0.05), showing that participants with different In-group Bias strength were differently affected by speaker Group Membership. Simple effect analyses revealed that while In-group Bias strongly modulated participants’ response bias with in-group labels (β = 8.60, SE = 2.55, *t* = 3.38, *p* < 0.001), this was only marginally so with out-group labels (β = 4.34, SE = 2.55, *t* = 1.71, *p* = 0.09). These findings show that participants differed in their response bias as a function of Group Membership and In-group Bias, so the more in-group biased they were, the more conservative they were in their in-group related decisions, as compared to out-group related decision (see Figure 5). In other words, they were more careful in attributing in-group words to any in-group speaker.

### Control Experiment

We hypothesized that the tendency to monitor speaker social identity was dependent on whether the affiliations were perceived as socially salient, or relevant. To test this, we ran a control experiment in which participants learned new words from Dutch native students attending two Italian universities, as part of an exchange program. In this experiment, group membership was not manipulated. Participants still learned from two groups of speakers, like in the Main Experiment, but here the speakers’ affiliations were supposed to be socially neutral because the speakers belonged to two foreign universities. Therefore, no differences were expected between the two groups. To control for potential visual dissimilarities between the logos used, participants performed the same perceptual matching task as in the Main experiment, responding to pairings involving the logos of the Italian universities. Similar to what we did in the Main experiment, we calculated an individual measure that in this case can be seen as an index of Visual Bias. This individual measure was entered in the statistical analyses.

#### Matching Trials

We ran a logistic mixed effects model with accuracy as the dependent measure and fixed effects for Affiliation (University1 vs. University2, reference level: University1), Visual Bias (centered), and their interaction. Block was included as covariate to control for potential confounds. We added per-participant and per-items random intercepts and by-participant slope for Affiliation.

Overall, participants’ accuracy in the matching trials was 57.52% (SD = 49.48) and above chance level, as confirmed by a one-sample *t*-test (i.e., 50%) (*t* = 5.84, *p* < 0.0001). Neither Affiliation, nor Visual Bias or their interaction significantly predicted accuracy (ps > 0.27). Participants’ accuracy was better in Block2 than in Block1 (β = 0.28, SE = 0.11, *z* = 2.51, *p* < 0.05).

#### Mismatching Trials

We ran a logistic mixed model analysis with fixed effects for Mismatch Type (Within- vs. Between-affiliation, reference level: Within), Affiliation (University1 vs. University2, reference level: University1), Visual Bias (centered continuous measure), and their interaction terms. We added Block as covariate, per-participant and per-item random intercepts and by-participant slopes for Affiliation and Mismatch Type.

Overall, participants’ accuracy on mismatching trials was 69.37% (SD = 46.10) and above chance level (i.e., 50%), as confirmed by a one-sample *t*-test (*t* = 39.53, *p* < 0.0001). Generally, participants were more accurate in the between–affiliation mismatches than in the within–affiliation mismatches (β = 0.20, SE = 0.07, *z* = 2.64, *p* < 0.01; mean = 70.55%, SD = 45.59 and mean = 68.18%, SD = 46.58, respectively), indicating that even the irrelevant social affiliations were encoded to some degree. Participants were also more accurate in Block2 than in Block1 (β = 0.92, SE = 0.05, *z* = 18.00, *p* < 0.0001; mean = 78.14%, SD = 41.33 and mean = 60.73, SD = 48.84, respectively). None of the other main effects or interactions resulted significant (ps > 0.16), showing that, unlike the modulating effect of in-group bias in the Main Experiment, participants’ memory for speaker-label pairings was not modulated by Visual Bias.

#### D-Prime and C Values

To be consistent, we also performed analyses over d-prime and C values, as we did in the Main experiment. Crucially, we did not expect any differences between the two academic affiliations. We calculated two d-prime values and two C values per participant for the two affiliations separately. We ran two linear mixed-effect models with either d-prime or C values as the dependent variable and Affiliation University1 vs. University2, reference level: University1), Visual Bias (centered continuous measure), and their interaction terms. The models included per-participant random intercepts.

**FIGURE 6:**
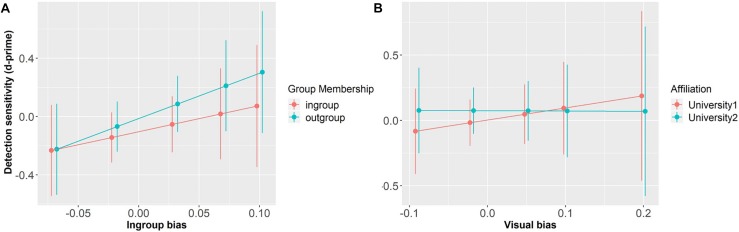
Detection sensitivity as a function of Group Membership and In-group Bias (centered) **(A)**, and as a function of Affiliation and Visual Bias (centered) **(B)**. Error bars represent standard errors.

The model that explored the relationship between individual d-prime and the independent variables showed no significant main effects or interactions (ps > 0.67), suggesting that participants’ sensitivity was not modulated by speaker Affiliation or their Visual Bias, nor the interaction between them (see Figure 6B).

**FIGURE 7:**
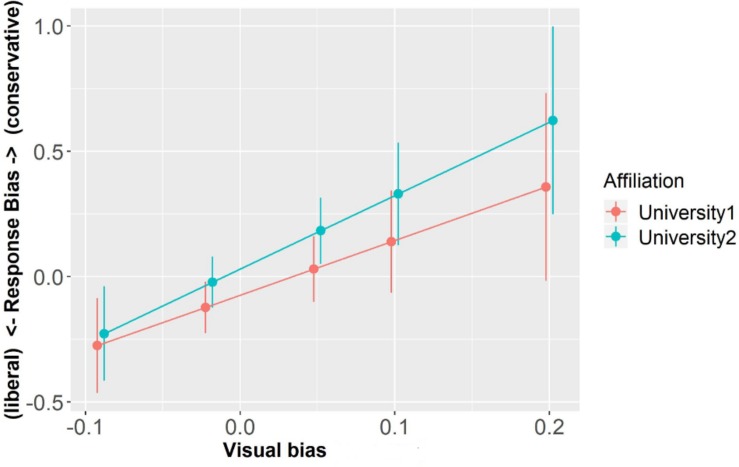
Response bias as a function of Affiliation and Visual Bias (centered). Error bars represent standard errors.

Similarly, the model exploring C values showed no significant main effect of Visual Bias or interaction (ps > 0.24). There was a marginal effect of Affiliation (β = 0.11, SE = 0.06, *t* = 1.90, *p* = 0.06) with decisions made about University2 being numerically more conservative than decisions involving University1 (see Figure 7).

## Discussion

We used a novel word learning paradigm to test whether learners of new words monitored speakers’ social identity, such as their group and individual identity. Furthermore, we asked whether group membership status of the speakers and individual in-group biases of the learners affected the level of detail of speaker-specific information encoded in the novel lexical representations. We additionally performed a control experiment and ensured that the patterns found in the Main experiment were indeed a reflection of the social saliency ascribed to speakers’ group membership and not simply a consequence of the contrastive nature of our manipulation (i.e., teaching competing labels spoken by different groups of speakers).

In the test phase of the word learning task, participants’ source memory for the new words was tested in an alternative forced-choice task (i.e., yes/no) where they decided whether displayed speaker-label pairs matched or mismatched what they learned in the exposure phase. This task offered a proxy for investigating the level of detail of speaker-specific information in the novel representations. Results confirmed our prediction regarding the general tendency to encode in parallel both linguistic content and speakers’ social identity (i.e., speakers’ affiliation). This tendency was reflected in the fact that participants made more within-affiliation errors than between-affiliation errors, i.e., source memory confusion. This finding provides further support for models of word learning where linguistic units are encoded together with speaker-related information (exemplar models e.g., Hay et al., 2006a; Goldinger, 2007; Nielsen, 2011; see Drager and Kirtley, 2016, for a review).

Concerning our hypotheses about the effects of Group Membership and In-group Bias, the results revealed a more complex pattern than we had predicted. We had predicted that participants would encode in-group labels with a higher level of detail of speaker-specific information, as compared to out-group labels. This phenomenon was expected to be reflected in (a) a higher proportion of hit rates for matching in-group speaker-label pairs and (b) a higher proportion of correct rejections for within-affiliation in-group speaker-label pairs. Both effects were predicted to be positively modulated by the individual In-group Bias, so that the stronger the bias, the stronger the effects. We found that indeed participants with stronger in-group bias were better at correctly rejecting wrong in-group pairings (i.e., in the mismatching trials). However, when looking at the matches, the results revealed that those participants with stronger in-group bias were also more likely to miss matching in-group speaker-label pairs.

These seemingly contradictory results are hard to reconcile when relying only on accuracy (i.e., correct/incorrect). For this reason, we relied on signal detection theory measurements, such as d-prime and C values, to gain a deeper understanding of the phenomenon. These measures capture both hit rates and false-alarm rates for conceptually similar items and allow us to test whether participants’ detection ability and/or response bias differed for in-group vs. out-group speaker-label pairs. Results showed that participants’ detection sensitivity was not modulated by our social manipulations such that they were equally sensitive to in-group and out-group speaker-label pairings. On the other hand, the model exploring C values showed that the more in-group biased, the more conservative participants were in their decision (i.e., having a bias for “no” responses), and this was particularly applied to in-group related decisions. That is, participants’ in-group bias and speakers’ group membership influenced *how liberally* decisions were made, so that participants with stronger in-group bias were more careful in attributing in-group labels to any speaker. This pattern explains why participants’ in-group bias negatively predicted hit rates and positively predicted correct rejection rates: the stronger the in-group bias, the more likely participants responded “no” to in-group speaker-label pairs.

How do our findings reconcile with the initial predictions and with previous literature? While previous studies showed that source memory was more accurate for information related to in-group membership, compared to information related to out-group membership (e.g., Hugenberg et al., 2010; Greenstein et al., 2016), in the current study we showed that the scenario can be more complex. Participants with a stronger bias were more accurate at correctly rejecting mismatches involving in-group labels, but they were also more likely to miss in-group matches. Looking closely at these patterns, we could deduce, and confirm with our analyses, that it was participants’ response bias that was mainly affected by our social manipulation of group membership, and by participants’ in-group bias. Participants with stronger in-group bias were in fact more cautious when attributing in-group labels to any speakers.

Our results resemble previous findings by Castano et al. (2002), who investigated if high vs. low in-group identifiers differed in their decision preferences when they had to categorize ambiguous faces as either in-group (i.e., Northern Italians) or out-group (i.e., Southern Italians) members. They found that participants that strongly identified with their in-group membership were less likely to classify a target face as in-group member, as compared to participants with a lower in-group identification score (see Yzerbyt et al., 1995; Blascovich et al., 1997; for similar results). The authors claimed that such a pattern was supportive of the *In-group overexclusion hypothesis* (Leyens and Yzerbyt, 1992), which states that when people are in doubt about classifying targets as either in-group or out-group, they tend to exclude them from their in-group. Such a hypothesis seems to apply to our dataset as well where participants with stronger in-group bias were more conservative when attributing in-group labels to speakers.

We consider why it is that learners’ in-group bias and speakers’ group membership status might lead to differences in response preferences, but not in detection sensitivity, as we had predicted. In other words, what might it mean that an individual with strong in-group bias is selectively more conservative when making a decision that involves her in-group membership? Originally, we had predicted group membership and in-group biases to play a role during the *encoding* of novel words, leading to in-group representations with more highly detailed speaker-specific information, as compared to out-group representations. The lack of modulation on the detection sensitivity measure by these social variables suggests that in-group and out-group labels did not differ in how they were encoded. Instead, we found a significant Group Membership × In-group bias effect on response bias, so that the stronger the in-group bias, the more conservative participants’ responses were in relation to in-group labels, but not in relation to out-group labels.

We believe that these differences in decision bias might reflect asymmetries during retrieval processes for in-group related episodic events, as compared to out-group related events. Previous research has shown that response bias acts during memory retrieval processes (Windmann et al., 2002) and depends on criterion setting functions of the prefrontal cortex (Schacter et al., 1998; Swick and Knight, 1999; Miller et al., 2001). During recognition decision-making processes, this brain region is considered to be involved in initiating, monitoring and controlling item-retrieval from memory to maintain a description of the information being sought and actively inhibit memory traces that do not match this description (Buckner, 1996; Fletcher et al., 1998; Wagner et al., 1998; Henson et al., 1999; Tomita et al., 1999). Therefore, Windmann et al. (2002) suggest that differences in response bias, especially when independent of the accuracy of the memory, can be explained by the fact that decision makers differ in what they prioritize in the task (i.e., the detection of matches or mismatches).

In light of this evidence, our findings might reflect differences in recognition threshold for in-group vs. out-group memory traces. During the decision processes, the inhibitory system of those participants who were more in-group biased was activated to a larger extent to avoid creating false positives and attributing in-group information to any source. Attributing in-group labels to incorrect speakers might have been perceived as more hurtful than missing the detection of correct in-group speaker-label pairs, as the in-group overexclusion hypothesis states. If this was indeed the case, these findings would validate the claim that in-group membership information recruits the control system to a larger degree than out-group membership does, as has been previously suggested (Meissner et al., 2005; Van Bavel and Cunningham, 2012). Furthermore, such a response bias could contribute to the effect known as out-group homogeneity in face recognition and categorization tasks (Castano et al., 2002), where new out-group faces produce more false alarms than new in-group faces do, supporting the claim that out-group members are perceived as more homogeneous.

Of course, it is important to replicate the present novel findings using different groups of speakers and different tasks, as to ensure that these effects and biases do not reflect poor recognition and/or high cognitive load in general. While the analyses revealed that participants’ accuracy was above chance level, it was still relatively low. Note that participants learned about the affiliations of the speakers during the word learning task, by seeing the faces of the speakers together with the logos of the supposed academic affiliations. This means that during the source memory test, they were potentially retrieving from their memory multiple pieces of information (e.g., speaker’s affiliation, label’s source). On that point, it is worth mentioning that even though in-group trials included a logo that might be more familiar than the out-group logo, as it is participants’ own university logo, participants did not exhibit superior memory for in-group items. Future studies should test whether our finding replicates when the source memory task is simplified, for instance, by participants learning the group membership status of speakers in an earlier experimental session, and in a more natural way (e.g., by listening to speakers referring to their university lives).

Similarly, to gain a deeper understanding of how speakers’ group membership and individual in-group biases influence language learning, it would be important to test whether source memory (i.e., the speaker) and item memory (i.e., the word) are equally affected by these social factors. While in this study we investigated the encoding of context-related information in the representations of novel words, and tested if its specificity was modulated by group membership and individual in-group biases, further research should test whether these factors influence the linguistic component of the representations, too. According to our general hypotheses, labels learned from in-group speakers would be easier to remember than words learned from out-group speakers.

If these patterns are substantiated, they will have far-reaching implications for theories of language learning and processing, as well as theories concerning prejudice and stereotyping. For instance, the results suggest that interlocutors’ group membership status and listeners’ individual biases may influence how likely newly acquired information is to be generalized to other interlocutors. In particular, for in-group speakers, listeners with a strong in-group bias appear to be more cautious when attributing in-group related information to other speakers, preventing over-generalization, whereas speakers with low in-group bias may be more liberal in their generalizations. One may wonder whether this greater caution relates to social stereotypes as well. It is well known that people tend to homogenize out-group members whereas they are aware of the heterogeneity of their own in-group. It would be interesting to examine to what degree such findings relate to the findings from this study about individuals’ greater cautiousness in attributing information to in-group compared with out-group members.

Further research should explore more how social characteristics that are ascribed to both speakers and contexts during language processing, and information processing more generally, influence encoding and storage, and how these, in turn, affect decision processes during memory retrieval. Such research would shed further light on the intersection between memory and processing, including language processing, and, importantly, how this intersection is influenced by the social properties of the input.

## Data Availability

The datasets generated for this study are available on request to the corresponding author.

## Ethics Statement

Human Subject Research: The studies involving human participants were reviewed and approved by Ethics committee of the Social Sciences department of the Radboud University Nijmegen (project code: ECSW2014-1003-196). The patients/participants provided their written informed consent to participate in this study.

## Author Contributions

SI organized the database, performed the statistical analysis, and wrote the first draft of the manuscript. All authors contributed to the conception and design of the study, wrote sections of the manuscript, and contributed to the manuscript revision, read, and approved the submitted version.

## Conflict of Interest Statement

The authors declare that the research was conducted in the absence of any commercial or financial relationships that could be construed as a potential conflict of interest.

## Footnotes

1 Due to copyright issues, none of the pictures of the gadgets in the example corresponds to actual stimuli, but they provide a good approximation of the type of stimuli we used.

2 To ensure that the patterns of results were comparable across both testing blocks, we also ran a mixed-effect model where response accuracy was modeled by Group Membership (In-group vs. Out-group, reference level: In-group), In-group Bias (centered continuous predictor), Block (Block1 vs. Block2, reference level: Block1) and their interactions. We added per-participant and per-items random intercepts and a by-participant slope for Group Membership. Results from this analysis showed that neither the main effect of Block (*p* = 0.37) nor its interactions with the other variables (ps > 0.16) significantly predicted response accuracy.

3 To calculate C and d-prime values, we firstly followed Macmillan and Creelman (2004) and converted 0 values in False Alarms to 1/2N and 1 values in Hit rates to 1-1/2N. Next, we subtracted the *z*-scored False Alarms rate from the *z*-scored Hit rate. C values, were calculated using the following formula: (−0.5) × (*z*-scored (Hit_Rate) + *z*-scored (False_Alarms rate)).
