# Supplementary material for: How In-Group Bias Influences Source Memory for Words Learned From In-Group and Out-Group Speakers
Source: Front Hum Neurosci. 2019 Sep 12;13:308. doi: 10.3389/fnhum.2019.00308 (PMC6751324; doi:10.3389/fnhum.2019.00308)
Supplement: Supplementary file 1 [file Table_1.DOCX]

# Appendices

### Appendix 1: Norming of labels and images for word learning

The aim of the norming study was to select the object images and labels for the word learning task, such that target objects would have at least two equally good labels

To do so, a two-phase norming study was carried out. In each phase, a different group of Dutch university students completed an online survey hosted on LimeSurvey ([LimeSurvey Project Team and Schmitz, 2012](http://www.sciencedirect.com/science/article/pii/S0264837714001902#bib0165)). Participants received either university-credit or monetary compensation.

1st phase: Written picture naming

Eighteen volunteers (12 female; age mean= 20.89, SD= 1.71) participated. They saw 120 images of uncommon gadgets collected from the internet (e.g., a corn peeler) and provided potential labels for them. After data collection, three Dutch native speakers corrected misspelt forms and excluded redundant forms , as well as regionalisms, non-Dutch forms, and inaccurate names (i.e., if the object was confused with something else). The majority of the elicited labels were nominal compounds with a semantic head (on the right, as common in Dutch), which refers to the action or the purpose of the gadget (e.g., peeler), and another noun (or compound) referring to the object that receives the action (e.g., corn). To ensure competing labels were comparable, labels not following this structure were excluded at this stage.

At the end of this process, 116 out of the initial 120 items were selected. Each gadget elicited between three and fourteen alternative labels.

2nd phase: Goodness-of-fit ratings

During the second phase, 40 university students (33 female; age mean=19.67, SD= 1.96) were recruited for a new online survey. The aim of the survey was to test how well the given labels described the gadgets. The 116 objects selected in the previous phase were pre-randomized and divided into three blocks. Six different versions of the survey were created in order to counterbalance the presentation order of the blocks. Each object was individually presented along with all the given labels. Participants had to rate how well each label described the item on a 1-to-7 scale (1 representing inappropriate, i.e., *ongeschikt*, and 7 being the perfect name, i.e., *perfecte benaming*). Participants were instructed that several labels for a specific item could receive the same value if they were equally good.

Next, paired t-tests were performed on the ratings of all possible pairwise combinations of labels for the same objects. Once we identified pairs which did not statistically differ in ratings (p>.05), we compared the frequency of their nominal constituents using SUBTLEX-NL (Keuleers, Brysbaert & Boris, 2010), to ensure they do not significantly differ in frequency either. For fillers, we selected items with one label that was rated as significantly better than all the other options (paired t-tests, ps<.05).

Forty-one target and 43 filler items fulfilled the requirements. To fulfill the requirements of another study that used the same stimuli, we also ensured that items’ familiarity is not judged to depend on level of education. Twelve target items and 12 fillers met these criteria.

### Appendix 2: Perceptual Matching Task results

#### Analyses over RTs

Prior to analyses, trials with incorrect responses or with RTs faster than 200ma or slower than 2100ms were excluded. For these confirmatory analyses, we selected only matching trials (i.e., in which the logo of the university was displayed with the associated geometrical shape) which referred to the in-group university and the out-group university used in the study (i.e., ROC Nijmegen). We then performed an outlier removal procedure by removing trials with RTs 2.5 SDs or higher from the mean per condition, per participant. The resulting dataset was analyzed using linear mixed-effect model in which log(10)-transformed RTs were predicted by the fixed effect for Group Membership (In-group vs Out-group, reference level: In-group). We added per-participant random intercept and by-participant random slope for Group Membership. Results confirmed the usual trend for this task: participants were faster at recognizing in-group-related associations than out-group-related associations (beta=-0.01, SE=0.003, t=-5.03, p<0.0001).

#### Analyses over Accuracy

Prior to analyses, we selected only matching trials (i.e., in which the logo of the university was displayed with the associated geometrical shape) which referred to the in-group university and the out-group university used in the study (i.e., ROC Nijmegen). Responses’ accuracy on a single trial were analyzed using logistic mixed-effect model with a fixed effect for Group Membership (In-group vs Out-group, reference level: In-group). We added per-participant random intercept and by-participant random slope for Group Membership. Results confirmed the usual trend for this task: participants were better at recognizing in-group-related associations than out-group-related associations (beta=0.4, SE=0.1, t=3.17, p<0.01).
